# Safety and Tolerability of HemoHIM: A Randomized, Placebo-Controlled, Double-Blind, and Parallel Clinical Trial in Healthy Human Volunteers

**DOI:** 10.4014/jmb.2503.03041

**Published:** 2025-07-25

**Authors:** Ji-won Seo, Jun-Hyun Bae, Ju Gyeong Kim, Su-Bin Bak, Gyoung-Deuck Kim, Wook Song

**Affiliations:** 1 Health and Exercise Science Laboratory, Department of Physical Education, Seoul National University, Seoul 08826, Republic of Korea; 2 Institute of Sports Science, Department of Physical Education, Seoul National University, Seoul 08826, Republic of Korea; 3 Muscle Biology Laboratory, School of Kinesiology, University of Michigan–Ann Arbor, MI 48109, United States of America; 4 Food Science R&D Center, Kolmar BNH Co., Ltd, Seoul 30003, Republic of Korea; 5 Institute on Aging, Seoul National University, Seoul 08826, Republic of Korea

**Keywords:** HemoHIM, safety, blood and urine tests, bioactive compounds, LC-MS/MS analysis

## Abstract

HemoHIM is a mixed extract derived from the roots of oriental medicinal herbs. Despite many studies on HemoHIM’s antioxidant properties and its effect on immune functions, fatigue reduction, and exercise performance, published data regarding its physiological safety in humans are lacking. Therefore, this study aimed to investigate the safety profile of HemoHIM in humans. The samples were analyzed using liquid chromatography–tandem mass spectrometry (LC-MS/MS) with ultra-high-performance LC coupled to an Orbitrap MS. Data processing and compound identification were performed through nontargeted screening using multiple spectral databases. Ninety-six healthy adults were recruited in the clinical study. On visit 1, screening was conducted to assess eligibility. On visit 2, participants were randomly assigned to either the HemoHIM group (*n* = 48) or the placebo group (*n* = 48), and they consumed 40 g/day either HemoHIM or placebo supplements twice daily for 8 week. Participants visited the laboratory four times for body composition, compliance, dietary intake, vital signs, blood and urine biomarker, and adverse event assessment. Several bioactive compounds were successfully identified using LC–heated electrospray ionization–Orbitrap–MS/MS, including chlorogenic acid, paeoniflorin, decursin, and nodakenin. No clinically significant changes were found in the safety test of vital signs, blood and urine biomarkers, and adverse events. Based on these findings, HemoHIM could be a safe and beneficial natural compound mixture for human use.

## Introduction

HemoHIM is an extract derived from a blend of roots of the oriental medicinal herbs *Angelica gigas* Nakai (Apiaceae), *Cnidium officinale* Makino (Apiaceae), and *Paeonia lactiflora* Pallas (Paeoniaceae) [1]. *Angelica* radix, the root of *A. gigas*, has been shown to improve exercise performance and improve fatigue in mice [2]. *Cnidium* rhizoma, the rhizome of *C. officinale*, promotes muscle recruitment and accelerates balanced metabolism [3]. A mixture containing *Paeonia* radix, the root of *P. lactiflora*, has been associated with reduced blood lactate level postexercise in basketball players [4]. These extracts were initially selected to formulate a supplement for enhancing antioxidant and immune function, resulting in the creation of HemoHIM as a health-promoting functional food. Consequently, HemoHIM has demonstrated effectiveness not only in antioxidant [5, 6] and immune functions [7] but also in improving fatigue [5, 8] and enhancing exercise performance [8]. Additionally, a recent study found no toxicity or genotoxicity in rats, providing evidence to support the safety of HemoHIM [9].

Despite extensive research on the functional benefits of HemoHIM, its physiological safety profile in humans remains limited and insufficiently characterized. Our research group has previously published findings demonstrating HemoHIM's efficacy in alleviating fatigue and enhancing exercise performance in human subjects [8]. In the present study, we aimed to conduct a comprehensive reassessment of HemoHIM's safety in healthy adults using advanced analytical techniques, including liquid chromatography–tandem mass spectrometry (LC-MS/MS), along with an in-depth examination of variables pertinent to physiological safety.

## Materials and Methods

### Study Design

**Fig. 1:**
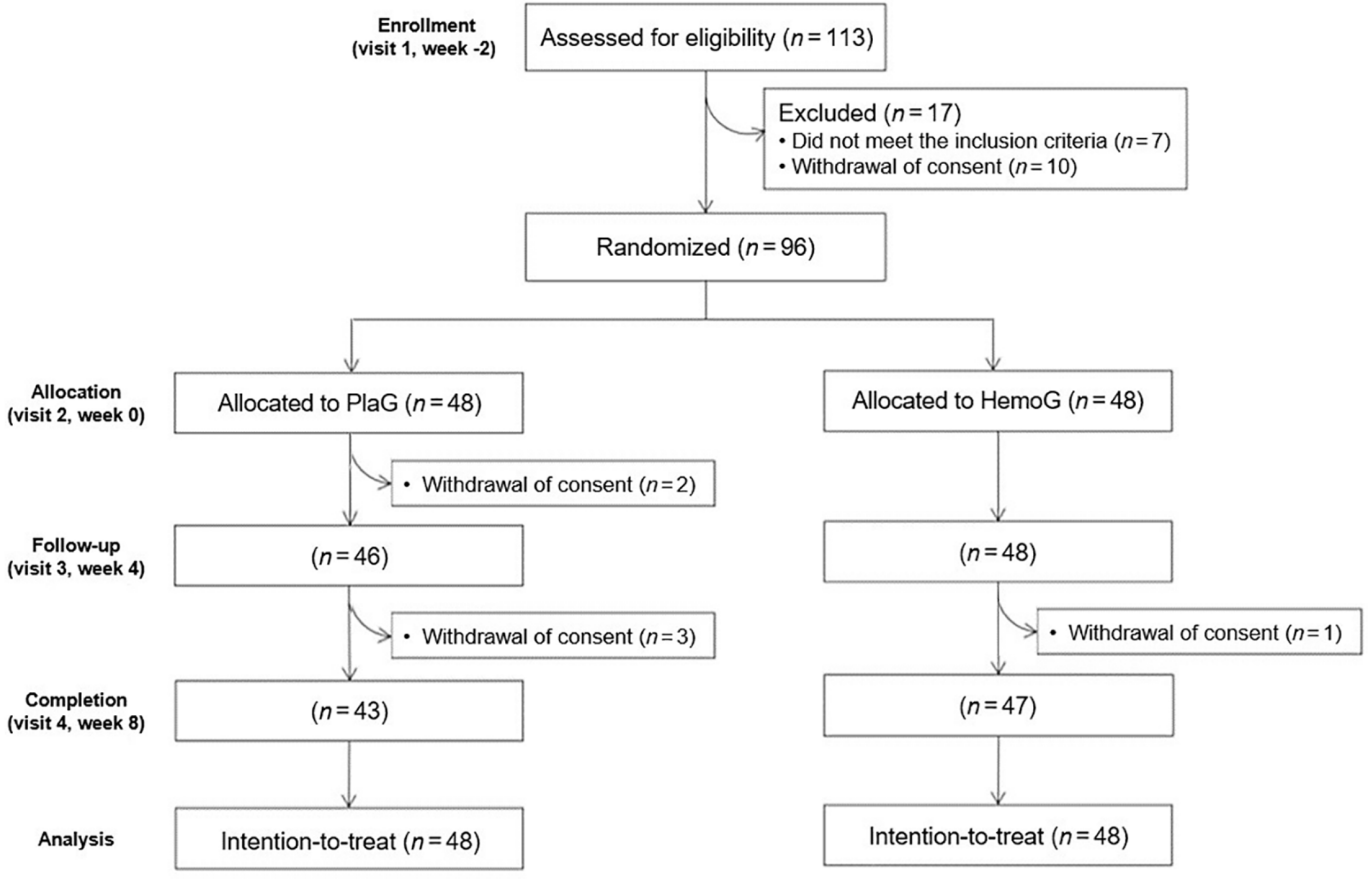
CONSORT flow diagram of the participants. PlaG, placebo group; HemoG, HemoHIM group.

This study was designed as a randomized, placebo-controlled, double-blind, and parallel clinical trial. Eligibility screening was conducted at visit 1 (week -2). Participants who met inclusion criteria were randomly assigned to the HemoHIM group (HemoG) or the placebo group (PlaG) at visit 2 (week 0) after a 2-week run-in period. Each participant consumed HemoHIM or placebo supplements twice daily for 8 weeks. They visited the laboratory a total of four times during the whole study period (Fig. 1). Assessments, including body composition, vital signs, dietary intake, blood and urine biomarkers, and adverse events, were conducted at visits 2 (week 0) and 4 (week 8). Supplement compliance was monitored at visits 3 (week 4) and 4 (week 8).

### Participants Eligibility Criteria

Sample size calculation was based on a previous research [10]. The minimum number of subjects required for this study, calculated using the G*Power 3.1 (Heinrich Heine University, Germany) was 80, with 40 persons/group.

Ninety-six healthy adults (aged 30-59 years; body mass index [BMI], 18.6-29.9 kg/m^2^) were recruited. Participants signed a written consent form. Those with excessive alcohol consumption (>210 g/week for men and >140 g/week for women), those with smoking or drug addiction, those with diseases or using disease-related medicines, those who were continuously taking herbal medicines or supplements within 1 month prior to the first visit, those who were pregnant or breastfeeding, and those participating in other clinical trials within 1 month prior to the first visit were excluded.

### Randomization and Blinding

Participants meeting the selection criteria were assigned numbers based on their order of registration at visit 2. They were then randomly assigned to the HemoG or PlaG according to a computer-generated random number list prepared by an independent third party prior to the commencement of the study. Block randomization was used to assign participants to the groups in a 1:1 ratio, with efforts to make the male-to-female ratio between groups as close as possible.

This study used a double-blind design, ensuring that both the participants and the researchers remained unaware of the group assignments until the conclusion of the study.

### Interventions

Each participant consumed 40 g/day of either HemoHIM or placebo in a concentrated liquid form twice daily (morning and evening) for 8 weeks from visits 2 to 4. The HemoHIM supplement contained 50% HemoHIM extract, 18.7% purified water, 15% cyclodextrin syrup, 15% starch syrup, 0.1% sodium alginate, 0.2% xanthan gum, 1.0% lactose mixed powder, 0.001% herbal flavor, and 0.001% cacao color. The placebo supplement contained 0% HemoHIM extract, 77.1% purified water, 12% starch syrup, 0.1% sodium alginate, 10.0% lactose mixed powder, 0.2% herbal flavor, 0.01% cacao color, 0.4% guar gum, and 0.1% microcrystalline cellulose.

During the study, participants were instructed to maintain their usual diet and lifestyle. Specifically, they were prohibited from consuming foods containing the same raw materials as HemoHIM (*A. gigas* Nakai, *C. officinale* Makino, and *P. lactiflora* Pallas), herbal medicines, and health functional foods. They were also advised to eat the same menu the day before each visit and to abstain from alcohol and caffeine 24 h prior to each visit.

### LC-MS/MS and Data Processing

**Sample preparation.** The sample was dissolved in 80% methanol and then centrifuged at 13,000 rpm for 15 min at 4°C to precipitate impurities (Centrifuge 5425 R; Germany). The supernatant was filtered through a 0.22-μm polyvinylidene fluoride membrane filter and transferred to a vial for analysis. All solvents used for MS analysis were of MS grade. Prepared samples were stored at 4°C and analyzed within 24 h to prevent degradation.

**LC conditions.** LC was performed using a Vanquish ultra-high-performance LC system (Thermo Fisher Scientific, USA) equipped with a Thermo Hypersil GOLD AQ C18 column (1.9 μm, 2.1 × 100 mm). A gradient elution was applied to separate components according to their polarity using solvent A (water containing 0.1% formic acid) and solvent B (acetonitrile containing 0.1% formic acid). The column oven was maintained at 36°C, the flow rate was 0.3 ml/min, and the injection volume was 2 μl. The autosampler temperature was maintained at 4°C. UV spectra were recorded from 190 to 800 nm.

**MS conditions.** MC detection was performed on a Q Exactive Orbitrap mass spectrometer (Thermo Fisher Scientific) operating in both positive and negative ion modes with heated electrospray ionization (HESI). The capillary temperature was set to 320°C, and the probe heater temperature was 300°C. The sheath gas was nitrogen, with a flow rate of 40 units, and the auxiliary gas was helium, with a flow rate of 10 units. The source voltage was set to 3.5 kV in the positive ion mode and 2.8 kV in the negative ion mode. The scan range was 100-1500 *m/z*. The resolution was set at 70,000 FWHM at *m/z* 200 for MS1 scans and 17,500 FWHM for MS2 scans. The MS was calibrated daily using a calibration mixture provided by the manufacturer to ensure mass accuracy.

**Data processing.** MS data were processed using Compound Discoverer version 3.2 (Thermo Fisher Scientific). The mass range for data processing was set from 100 to 1500 *m/z*, with a retention time (RT) range of 0.5 to 20 min. Data alignment was performed with a minimum intensity of 2 × 10^7^, *m/z* tolerance of 10 ppm, and RT tolerance of 2 min. Detected features were grouped across all samples using the same *m/z* tolerance (10 ppm) and gap-filled where necessary. Compound identification was performed via nontargeted screening using the mzCloud database (https://www.mzcloud.org/). For compounds that remained unidentified after initial processing, further analysis was performed by searching the Metlin, MassBank, and MoNA databases and comparing MS2 fragmentation patterns and molecular weights.

**Data quality.** In this study, quality control procedures were based on solvent blanks and analyzed to identify and exclude background signals and contaminants. Additionally, quality control samples were prepared by pooling aliquots from all samples and analyzed every 10 injections to monitor system stability and reproducibility. Daily calibration of the MS was performed using a calibration mixture provided by the manufacturer to ensure mass accuracy.

### Measurements

**Anthropometric measures.** Participants’ sex and age were recorded. Height was measured using a manual stadiometer. Weight, BMI, body fat mass, and skeletal muscle mass were measured using bioelectrical impedance analysis (InBody720; InBody, Republic of Korea). Before the measurements, participants were instructed to empty their bladders, wear light clothing, and remove any metal items. Measurements were performed by the same researcher using the same machine, at the same time of day during each visit.

**Compliance.** Supplement compliance was calculated using the following equation: compliance (%) = {number of supplements consumed/(number of days to consume×2)}×100.

**Dietary intake.** In the period between each visit, participants’ dietary intake (total calories, carbohydrates, fat, protein, and sodium) was recorded on 2 weekdays and 1 weekend day (a total of 3 representative days) using a mobile application.

**Vital signs.** After participants rested for at least 10 min, their body temperature, blood pressure, and pulse rate were measured. These measurements were performed at the same time of day during each visit by the same researcher, using the same machine.

**Clinical laboratory tests.** Blood and urine samples were collected after participants fasted for more than 12 h. Blood tests included the following: white blood cell (WBC), red blood cell (RBC), hemoglobin (Hb), hematocrit (Hct), and platelet (PLT) counts; mean corpuscular volume (MCV); mean corpuscular hemoglobin (MCH); mean corpuscular hemoglobin concentration (MCHC); neutrophils, lymphocyte, monocyte, eosinophil, and basophil levels; aspartate aminotransferase (AST), alanine aminotransferase (ALT), glucose, creatinine, blood urea nitrogen (BUN), total protein, albumin, total bilirubin, alkaline phosphatase (ALP), and uric acid levels. Urine tests included the following: specific gravity, pH, nitrite, protein, glucose, ketone, urobilinogen, and bilirubin levels.

**Adverse events.** Participants were asked to voluntarily report any adverse events at any time. At each visit, the researchers also interviewed participants to confirm if any adverse events occurred.

### Statistical Analyses

An intention-to-treat analysis was conducted, which included all registered participants. Nonnormal distribution were transformed to a normal distribution before analysis. Baseline characteristics were compared between groups using Student's *t*-test for continuous variables and the chi-square test for categorical variables. Compliance between groups was analyzed by Student’s *t*-test. Continuous variables were analyzed by the linear mixed-effects model to investigate the effects of group, week, and group×week interaction. Categorical variables were presented as the number of participants and analyzed using Fisher's exact test for between-group comparisons and McNemar's test for within-group comparisons. SAS version 9.4 (SAS Institute, USA) was used to analyze the data, and statistical significance was set at 5%.

### Ethics Statement

This study was reviewed and approved by the institutional review board of Seoul National University (IRB no. 1912/003-003). The trial was conducted in accordance with the ethical principles of the Declaration of Helsinki. Participants who agreed to participate in this study provided written informed consent.

## Results

### Participants

A total of 96 participants were enrolled and randomly assigned to the HemoG or PlaG (*n* = 48, respectively). During the 8-week intervention period, 6 people withdrew consent, and 90 people completed the study (Fig. 1). The average age of the participants in the HemoG was 43.4 ± 1.2 years and that of the participants in the PlaG was 45.0 ± 1.2 years. Both groups had an equal sex distribution, with no significant differences in baseline characteristics (Table 1). Additional analyses by sex also showed no differences between groups in either male or female (Table S1). The compliance rate was 99.4 ± 1.0% for the HemoG and 99.4 ± 0.9% for the PlaG, with no significant difference between groups (*p* = 1.000).

### LC-MS/MS Analysis

**Results of chromatographic separation.** The chemical composition of the sample was analyzed using LC-MS/MS based on the HESI-Orbitrap technique. The resulting chromatograms and MS data enabled the identification of key compounds. The LC chromatogram displayed distinct peaks corresponding to the identified compounds' RTs. Chlorogenic acid (CGA) was detected at an RT of 6.65 min, whereas paeoniflorin was identified at approximately 7.62 min. Nodakenin and nodakenin derivatives were detected between 8.45 and 8.97 min. Decursin, the final peak identified, was detected at 14.50 min.

**Results of compounds identified by LC-MS/MS.** MS was performed in both positive and negative ion modes using HESI with the Orbitrap mass analyzer. Fragmentation patterns were obtained using MS/MS, allowing structural elucidation based on MS2 data. In the negative ion mode, CGA ([M-H]-, *m/z* 353.088) and paeoniflorin ([M+FA-H]-, *m/z* 525.162) were among the primary identified compounds. In the positive ion mode, decursin ([M+H]+, *m/z* 329.138) and nodakenin ([M+H]+, *m/z* 409.149) were successfully identified based on their characteristic fragmentation patterns (Table 2).

### Safety

**Vital signs.** There were no significant differences in blood pressure and pulse rate within or between groups. However, body temperature decreased by 0.2°C at the posttest (week 8) compared with the pretest (week 0) in the PlaG, which was statistically significant (*p* = 0.015; Table 3).

**Clinical laboratory tests.** Blood test results (Table 4) revealed significant changes in RBC count between the groups (*p* = 0.029). There was no change in RBC count in the HemoG, whereas that in the PlaG decreased by 0.1 × 10^6^/μl. The MCHC values increased at posttest compared with pretest values in both groups (PlaG = 0.3 g/dl, *p* = 0.011; HemoG = 0.2 g/dl, *p* = 0.044). The total bilirubin levels in the PlaG decreased by 0.09 mg/dl (*p* = 0.022). The ALP levels in HemoG increased by 4.9 U/l over 8 wk, with significant differences within and between groups (within-group *p* < 0.001; between-group *p* = 0.009). However, all changes were within normal ranges.

Urine test results showed no significant differences within or between groups (Table 5).

**Adverse events.** No adverse events were reported in the HemoG. One participant in the PlaG reported an adverse event of difficulty sleeping, but the symptoms were mild and unrelated to supplement intake (Table 6).

## Discussion

This study evaluated the safety of an 8-week HemoHIM intake regimen in healthy adults aged 30-59 years. The results demonstrated the successful identification of several bioactive compounds using LC-HESI-Orbitrap-MS/MS, including CGA, paeoniflorin, decursin, and nodakenin. The presence of CGA, a well-known antioxidant and anti-inflammatory compound, suggests that the sample may have potential therapeutic properties. Furthermore, the detection of paeoniflorin highlights its neuroprotective potential, consistent with previous findings that suggest these compounds play a key role in regulating inflammatory pathways. Notably, decursin, which is implicated in anti-inflammatory processes, was identified.

Our results indicated that CGA was one of the compounds identified by LC-MS/MS analysis (Table 2). A previous study reported that CGAs are prevalent in many herbal infusions, with yerba mate, assa-peixe, white tea, winter’s bark, green tea, and elderflower showing the highest concentrations [12]. This finding aligns with the reported health benefits of CGA, notably its potential role in diabetes mellitus and metabolic syndrome [13] and its use in traditional Chinese medicine [14]. The specific mechanism of CGAs indicates that high-dose CGAs induced inflammation and oxidative stress in animal models, potentially due to an imbalance between oxidant and antioxidant mechanisms [15]. Overall, this study found that HemoHIM contains CGA, a compound that possesses significant health benefits, such as antioxidant, anti-inflammatory, and antiviral properties.

Additionally, our results identified paeoniflorin as a key compound from the LC-MS/MS analysis (Table 2). Previous studies have successfully quantified paeoniflorin in various herbal preparations using LC-MS/MS [16]. Paeoniflorin exhibits antidepressive properties, with potential mechanisms related to neuroprotection, neurogenesis, and inflammation regulation [17]. Therefore, our results support the potential of HemoHIM for improving neurological health.

Our LC-MS/MS analysis also identified decursin and nodakenin as significant compounds (Table 2). Decursin, derived from *A. gigas* Nakai, shows promise in anticancer therapy. Studies have demonstrated its ability to induce apoptosis and inhibit proliferation in various cancer cell lines, including prostate and breast cancer cells [18,19]. Nodakenin, found in *Angelica koreana*, has been the subject of studies focusing on cognitive enhancement, showing the potential to improve memory and learning in animal models [20], possibly through the modulation of cholinergic neurotransmission and neuroprotective mechanisms. These studies reinforce the therapeutic potential of HemoHIM and support its continued investigation for use in herbal medicine and drug development.

In this study, we evaluated safety in humans via measurements of vital signs (systolic and diastolic blood pressure, pulse rate, and body temperature), blood tests (WBC, RBC, Hb, Hct, PLT, MCV, MCH, MCHC, neutrophils, lymphocytes, monocytes, eosinophils, basophils, AST, ALT, glucose, creatinine, BUN, total protein, albumin, total bilirubin, ALP, and uric acid), urine tests (specific gravity, pH, and nitrite, protein, glucose, ketone, urobilinogen, and bilirubin), and adverse events.

First, among vital signs (Table 3), body temperature significantly decreased in the PlaG after 8 wk, with no change in the HemoG. In this study, body temperature was measured on the forehead using a noncontact infrared thermometer, and the normal temperature on the forehead ranges from 34.7°C to 37.3°C [21]. Additional analyses by sex also showed no clinically significant changes (Table S2).

Second, in the blood test (Table 4), there was a significant difference in RBC count between the groups, which caused by a decrease in the PlaG but not in the HemoG, with values remaining within the normal range. MCHC levels increased in both groups at the posttest but remained within the normal range. Total bilirubin levels significantly decreased in the PlaG, but within the normal range. ALP levels increased posttest in the HemoG, with significant differences within and between the groups; however, these values were also within the normal range. Additional analyses by sex also showed no clinically significant changes (Table S3).

Third, there were no significant differences within or between the groups in urine test results (Table 5). Lastly, no adverse events were reported in the HemoG (Table 6).

Although there were changes in some variables in this study, experts, including medical doctors, deem these clinically insignificant, considering the absence of physical symptoms and the values of other variables.

It is crucial to note the limitations of the study. This study focused on healthy adults aged 30-59 years with a specific BMI range. Results may not generalize to populations outside these age and BMI ranges, such as younger adults, older individuals, or those with obesity or underweight conditions. Future studies should include participants across broader age ranges, BMI categories, and health conditions. The intervention lasted 8 wks, which may not be sufficient to determine the long-term effects and safety of HemoHIM supplementation. Conducting studies over a longer period (*e.g.*, 6 months to 1 year) could better evaluate the long-term effects, safety, and adherence to HemoHIM supplementation. Finally, since this study was conducted under well-controlled conditions, including daily dietary intake and physical activity, potential confounding by these factors could not be predicted.

In conclusion, based on the results of this study, HemoHIM is a mixture of natural materials that may provide functional benefits, and its safety has also been confirmed through 8-week daily intake in healthy adults.

## Supplemental Materials

Supplementary data for this paper are available on-line only at http://jmb.or.kr.



## Figures and Tables

**Table 1:** Baseline characteristics of the participants.

Variables	Placebo	HemoHIM
Age (years)	43.4 ± 1.2	45.0 ± 1.2
Sex (men/women)	12/36	12/36
Body weight (kg)	62.8 ± 1.5	62.4 ± 1.8
BMI (kg/m^2^)	23.3 ± 0.4	23.4 ± 0.4
Fat mass (kg)	18.2 ± 0.8	18.2 ± 0.8
Lean mass (kg)	44.6 ± 1.3	44.2 ± 1.6
Percent body fat (%)	29.1 ± 1.0	29.4 ± 1.1
Skeletal muscle mass (kg)	24.5 ± 0.8	24.3 ± 1.0
Dietary intake		
Energy (kcal/day)	1529.0 ± 44.8	1501.5 ± 43.1
Carbohydrate (g/day)	227.8 ± 7.2	220.9 ± 6.8
Protein (g/day)	58.0 ± 2.0	58.3 ± 1.9
Fat (g/day)	43.4 ± 1.9	43.5 ± 2.3
Sodium (mg/day)	3462.7 ± 178.2	3523.0 ± 147.3

Values are presented as mean ± standard error or n/n.

*p < 0.05 (Student’s *t*-test and chi-square test were used for continuous and categorical variables, respectively, to compare differences between groups).

**Table 2:** Results of the identification of major compounds using liquid chromatography–tandem mass spectrometry.

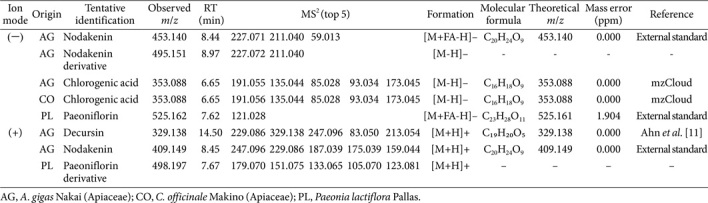

**Table 3:** Changes in vital signs over 8 week.

Variables	Placebo	HemoHIM
SBP (mmHg)		
Week 0	114.9 ± 1.6	112.2 ± 1.8
Week 8	112.7 ± 2.3	111.4 ± 1.6
DBP (mmHg)		
Week 0	75.9 ± 1.4	73.7 ± 1.4
Week 8	73.9 ± 1.8	75.0 ± 1.3
Pulse rate (beats/min)		
Week 0	78.8 ± 1.9	75.9 ± 1.4
Week 8	79.2 ± 2.0	76.7 ± 1.5
Body temperature (°C)		
Week 0	36.3 ± 0.1	36.1 ± 0.1
Week 8	36.1 ± 0.1*	36.2 ± 0.1

Values are presented as mean ± standard error.

SBP, systolic blood pressure; DBP, diastolic blood pressure.

*p < 0.05 (Linear mixed-effect model was used to analyze the difference within each group).

^†^*p* < 0.05 (Linear mixed-effect model was used to analyze the group×week interaction).

**Table 4:** Changes in blood biomarkers over 8 week.

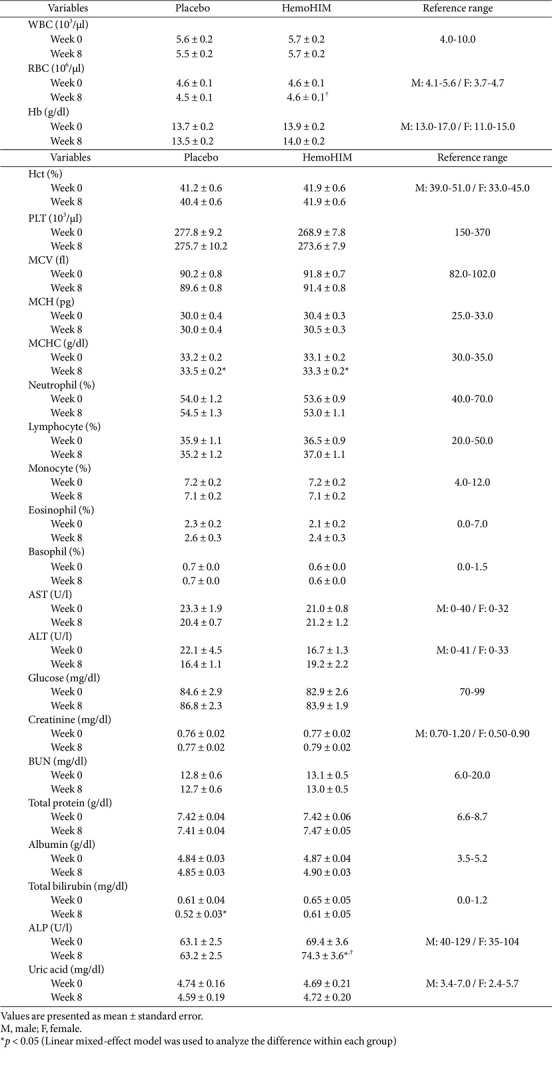

**Table 5:** Changes of urine biomarkers over 8 week.

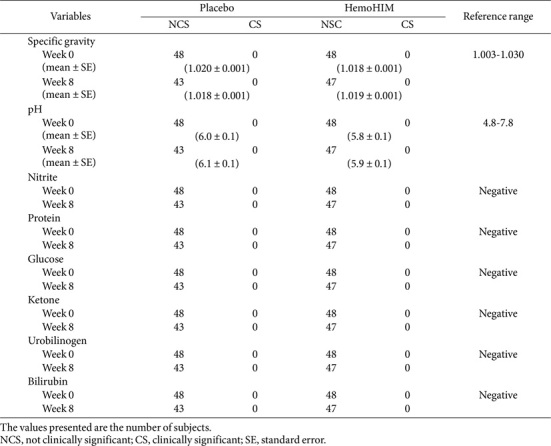

**Table 6:** Results of adverse events.

Variables	Placebo	HemoHIM
Adverse event case	1/1	0/0*
Symptoms		
Sleep discomfort	1/1	0/0*
Severity		
Mild	1/1	0/0*
Moderate	0/0	0/0
Severe	0/0	0/0
Relevance to supplements		
Certain	0/0	0/0
Probable	0/0	0/0
Possible	0/0	0/0
Unlikely	0/0	0/0
None	1/1	0/0*
Unknown	0/0	0/0

The values presented are the number of subjects/number of cases.

*p < 0.05 (Fisher’s exact test was used to compare differences in the number of subjects between the groups).
