# Supplementary material for: Safety and Tolerability of HemoHIM: A Randomized, Placebo-Controlled, Double-Blind, and Parallel Clinical Trial in Healthy Human Volunteers
Source: J Microbiol Biotechnol. 2025 Jul 25;35:e2503041. doi: 10.4014/jmb.2503.03041 (PMC12324994; doi:10.4014/jmb.2503.03041)
Supplement: Supplementary file 1 [file jmb-35-e2503041-supple.pdf]

J. Microbiol. Biotechnol. 2025. 35: e253041
https://doi.org/10.4014/jmb.2503.03041

S

Supplementary Tables

Table S1. Baseline characteristics of the participants by sex.

**Variables**	**Male**		**Female**	
	**Placebo**	**HemoHIM**	**Placebo**	**HemoHIM**
n	12	12	36	36
Age (years)	38.3±2.3	36.7±2.1	45.1±1.4	47.8±1.1
Body weight (kg)	76.4±1.8	76.8±3.6	58.6±1.3	57.6±1.2
BMI (kg/m^2)	25.4±0.5	24.7±0.8	22.6±0.5	22.9±0.4
Fat mass (kg)	17.1±1.2	15.9±1.6	18.6±0.9	18.9±0.8
Lean mass (kg)	58.6±1.4	60.9±2.9	40.0±0.6	38.7±0.6
Percent body fat (%)	22.5±1.3	20.5±1.7	31.3±1.0	32.4±0.9
Skeletal muscle mass (kg)	33.1±0.9	34.5±1.7	21.6±0.4	20.9±0.4
Dietary intake				
Energy (kcal/day)	1679.8±89.3	1561.7±67.4	1478.8±49.6	1481.5±53.0
Carbohydrate (g/day)	237.9±13.6	209.8±12.5	224.4±8.4	224.6±8.0
Protein (g/day)	65.1±3.1	64.7±3.9	55.7±2.3	56.2±2.1
Fat (g/day)	51.3±4.4	50.8±5.0	40.7±2.0	41.1±2.5
Sodium (mg/day)	3726.9±338.8	2997.5±250.6	3374.6±209.6	3698.2±169.6

Values are presented as mean±standard error.
Student's *t*-test was used to compare differences between groups.

Table S2. Changes in vital signs over 8 wk by sex.

Variables	Male		Female	
	Placebo	HemoHIM	Placebo	HemoHIM
SBP (mmHg)				
Week 0	124.0±2.8	117.2±3.0	111.9±1.7	110.5±2.1
Week 8	122.3±3.8	118.2±2.8	109.8±2.7	109.1±1.9
DBP (mmHg)				
Week 0	77.9±2.5	75.8±3.2	75.2±1.7	73.0±1.5
Week 8	77.5±3.7	79.4±2.3	72.8±2.1*	73.5±1.5
Pulse rate (beats/min)				
Week 0	73.8±3.5	71.4±3.0	80.5±2.2	77.4±1.5
Week 8	76.2±3.5	73.0±2.9	80.2±2.3	78.0±1.8
Body temperature (°C)				
Week 0	36.5±0.1	36.3±0.1	36.3±0.1	36.1±0.1
Week 8	35.8±0.1*	36.1±0.2†	36.2±0.1	36.2±0.1

Values are presented as mean±standard error.

SBP, systolic blood pressure; DBP, diastolic blood pressure.

*$p<0.05$ (Linear mixed-effect model was used to analyze the difference within each group).

$^{\dagger}p<0.05$ (Linear mixed-effect model was used to analyze the group×week interaction).

Table S3. Changes in blood biomarkers over 8 wk by sex.

Variables	Male		Female	
	Placebo	HemoHIM	Placebo	HemoHIM
WBC (10^3/μl)				
Week 0	5.1±0.1	6.4±0.4	5.8±0.2	5.5±0.2
Week 8	5.6±0.4	6.1±0.4	5.5±0.3*	5.6±0.2†
RBC (10^6/μl)				
Week 0	5.0±0.1	5.1±0.1	4.4±0.0	4.4±0.1
Week 8	5.0±0.1	5.2±0.1	4.4±0.0	4.4±0.1
Hb (g/dl)				
Week 0	15.6±0.2	15.6±0.3	13.1±0.2	13.3±0.2
Week 8	15.4±0.2	15.8±0.3	13.0±0.2	13.4±0.2
Hct (%)				
Week 0	46.1±0.5	46.6±0.7	39.6±0.5	40.3±0.6
Week 8	44.9±0.7	46.4±0.7	39.0±0.5	40.4±0.5
PLT (10^3/μl)				
Week 0	269.8±14.5	276.3±15.3	280.4±11.3	266.4±9.2
Week 8	265.3±12.4	275.1±14.5	278.8±12.7	273.1±9.4
MCV (fl)				
Week 0	91.6±1.3	91.6±0.8	89.8±1.0	91.9±1.0
Week 8	90.7±1.2	90.1±1.2*	89.3±1.0	91.8±0.9
MCH (pg)				
Week 0	31.0±0.3	30.6±0.4	29.6±0.5	30.3±0.4
Week 8	31.1±0.4	30.5±0.4	29.7±0.5	30.4±0.4
MCHC (g/dl)				
Week 0	33.9±0.3	33.4±0.2	32.9±0.2	33.0±0.2
Week 8	34.3±0.2	33.9±0.2	33.2±0.2*	33.1±0.2
Neutrophil (%)				
Week 0	52.9±2.5	54.6±1.7	54.4±1.4	53.3±1.1
Week 8	55.1±2.6	53.3±1.8	54.4±1.5	52.8±1.4
Lymphocyte (%)				
Week 0	36.7±2.3	35.1±1.8	35.6±1.3	37.0±1.1
Week 8	34.3±2.5	35.5±2.0	35.4±1.4	37.6±1.4
Monocyte (%)				
Week 0	8.0±0.4	7.0±0.5	6.9±0.3	7.3±0.3
Week 8	7.6±0.4	7.7±0.5†	7.0±0.3	7.0±0.2

Table S3. Changes in blood biomarkers over 8 wk by sex (continued). 18

Variables	Male		Female	
	Placebo	HemoHIM	Placebo	HemoHIM
Eosinophil (%)				
Week 0	1.9±0.4	2.8±0.6	2.5±0.3	1.9±0.2
Week 8	2.5±0.3	3.1±0.7	2.7±0.3	2.2±0.3
Basophil (%)				
Week 0	0.7±0.1	0.6±0.1	0.7±0.1	0.5±0.0
Week 8	0.6±0.1	0.6±0.1	0.7±0.0	0.6±0.1
AST (U/l)				
Week 0	23.6±2.6	22.5±2.1	23.2±2.4	20.6±0.8
Week 8	21.6±1.6	20.8±2.1	20.0±0.7	21.3±1.5
ALT (U/l)				
Week 0	20.8±3.7	19.0±2.6	22.5±5.8	15.9±1.5
Week 8	18.1±1.9	22.6±4.3	15.9±1.3	18.0±2.5
Glucose (mg/dl)				
Week 0	82.1±5.8	78.7±6.5	85.4±3.4	84.3±2.7
Week 8	80.6±3.4	77.1±3.9	88.6±2.8	86.2±2.1
Creatinine (mg/dl)				
Week 0	0.94±0.04	1.00±0.03	0.70±0.01	0.70±0.01
Week 8	1.00±0.00	1.02±0.03	0.71±0.02	0.71±0.01
BUN (mg/dl)				
Week 0	14.1±0.7	12.9±0.8	12.4±0.8	13.1±0.6
Week 8	15.2±1.1	12.9±1.1	12.0±0.7	13.0±0.5
Total protein (g/dl)				
Week 0	7.44±0.08	7.53±0.12	7.41±0.05	7.39±0.07
Week 8	7.45±0.07	7.60±0.09	7.40±0.05	7.43±0.06
Albumin (g/dl)				
Week 0	5.06±0.07	5.09±0.08	4.77±0.03	4.79±0.04
Week 8	4.97±0.08	5.05±0.06	4.82±0.03	4.85±0.03*
Total bilirubin (mg/dl)				
Week 0	0.70±0.10	0.85±0.10	0.58±0.04	0.58±0.05
Week 8	0.55±0.08	0.82±0.11	0.52±0.03	0.53±0.05
ALP (U/l)				
Week 0	68.1±4.7	73.9±2.9	61.4±2.9	67.9±4.7
Week 8	67.6±5.0	75.6±2.8	61.9±2.9	73.9±4.7*,†
Uric acid (mg/dl)				

Week 0	5.90±0.26	6.07±0.56	4.36±0.16	4.23±0.15
Week 8	6.01±0.34	6.04±0.45	4.16±0.16	4.27±0.16

Values are presented as mean±standard error.

*$p<0.05$ (Linear mixed-effect model was used to analyze the difference within each group).

†$p<0.05$ (Linear mixed-effect model was used to analyze the group×week interaction).
